# Malunion of the Acetabulum Treated by Intra-articular Buttress

**DOI:** 10.1155/2020/8361690

**Published:** 2020-07-06

**Authors:** D. Saragaglia, J. Gaillot

**Affiliations:** ^1^CHU Grenoble-Alpes Voiron Site, France; ^2^Grenoble Faculty of Medicine, France; ^3^Faculty of Medicine, France; ^4^CHU Grenoble-Alpes, France

## Abstract

Acetabulum malunions are extremely difficult to treat, and for many years, surgical indications have been dominated by total hip replacement. We treated a protruding acetabular malunion, 20 years ago, using an intra-articular buttress, by means of an allograft corresponding to a femoral head fragment which had been cryopreserved. The radiological and clinical result with this extended follow-up is quite remarkable, which has motivated us to present this original technique.

## 1. Introduction

Acetabulum malunions are extremely difficult to treat [1, 2], and for many years, surgical indications have been dominated by total hip replacement [3, 4, 5]. While this indication is perfectly justified in mature adults or where there is significant degenerative damage to the hip, it can be debatable in younger adults, especially when we hope to restore joint congruence, where there are no radiological signs of osteoarthritis. We recently saw a patient we had treated over 20 years ago with an intra-articular buttress, for a malunion with acetabular protrusion, and the quality of the result has motivated us to present this original technique.

## 2. Clinical Notes

**Figure 1:**
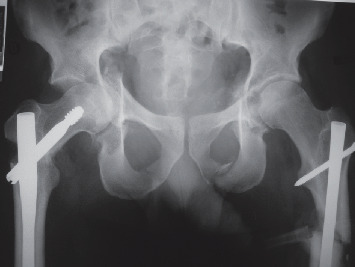
Initial state of the right acetabular fracture.

Mr. D., aged 31, was hospitalised urgently in late June 1999, in a teaching hospital 300 km from his home, following a polytrauma sustained in a motorcycle accident. The initial workup revealed a left haemopneumothorax, a ruptured spleen, bilateral acetabulum fractures with intrapelvic protrusion on the right, a bifocal right femur fracture, and a diaphyseal left femoral fracture. A haemostatic splenectomy was carried out immediately and the haemopneumothorax drained. The femoral fractures were nailed 2 days later, but no surgery was carried out on the right acetabulum. The patient was transferred to our facility 5 weeks after sustaining the initial injuries, to be closer to family, presenting acetabular protrusion of the right femoral head (Figure 1). Given the patient's age and the time elapsed since the injury, we decided to attempt conservative surgery to correct this unreduced fracture.

## 3. Surgical Procedure

**Figure 2:**
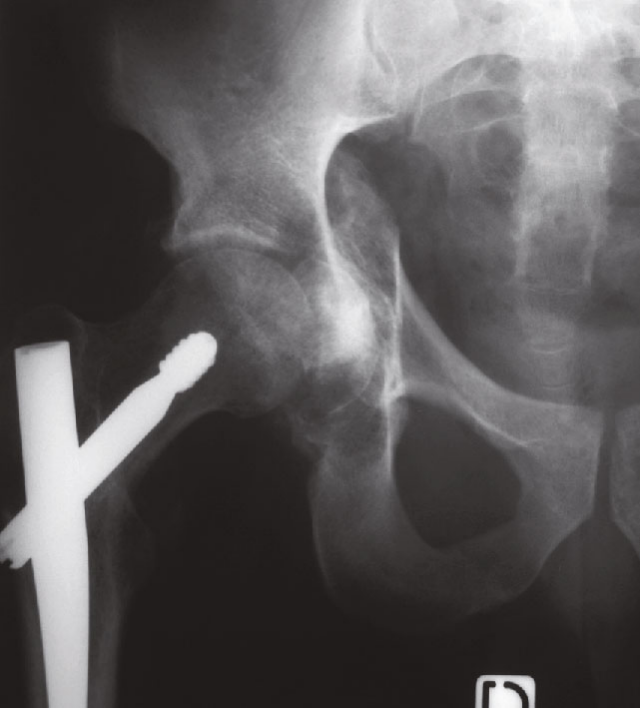
Postoperative X-ray of the intra-articular buttress perfectly correcting the femoral head acetabular protrusion.

The patient underwent surgery on 5 August 1999, 5 weeks after the initial accident. We decided to use a lateral approach, so as to access both acetabular columns simultaneously. The patient was positioned on a standard operating table, in lateral decubitus, with pubic and lumbosacral contact, and a purely lateral incision was made, with excision of the lateral scar from the long gamma nail implantation. This scar ran from the iliac crest to 15 cm below the tip of the greater trochanter. A 20 cm longitudinal opening of the fascia lata provided perfect exposure of the trochanteric region and the gluteus medius. Due to the presence of the nail, we decided to section the gluteus medius transversally, 2 cm above its trochanteric insertion, lifting the gluteal muscles, which were held back by 3 Steinman pins, nailed into the iliac wing. The gluteus minimus was also disinserted along the greater trochanter, fully exposing the anterior and upper parts of the articular capsule of the hip joint. To the rear, it was necessary to disinsert the upper pelvitrochanteric tendons, without touching the quadratus femoris. In order to penetrate into the joint, we carried out a periacetabular arthrotomy, followed by a proximal longitudinal incision along the axis of the femoral neck, and a disinsertion of the anterior capsular flap along the intertrochanteric line. This provided us with a perfect view of both columns as well as the joint cavity. Unfortunately, all attempts to reduce the fracture failed, as the fracture had consolidated at both the front and rear. When luxating the hip joint, we observed that the acetabular cavity was perfectly congruent with the femoral head and that simply pressing a compress deep into the acetabulum would restore this congruence. We therefore decided to replace this compress with a cryopreserved femoral head fragment from the bone bank, which we fitted to the bottom of the acetabulum and the femoral head, forming a self-stabilising intra-acetabular buttress (Figure 2). The image intensifier control was found to be perfect, and the incision was closed with 2 percutaneous drainage catheters in place, taking care to suture the capsule and sectioned tendons (gluteus medius and proximal pelvitrochanterics) thoroughly.

## 4. Postoperative Care and Results

**Figure 3:**
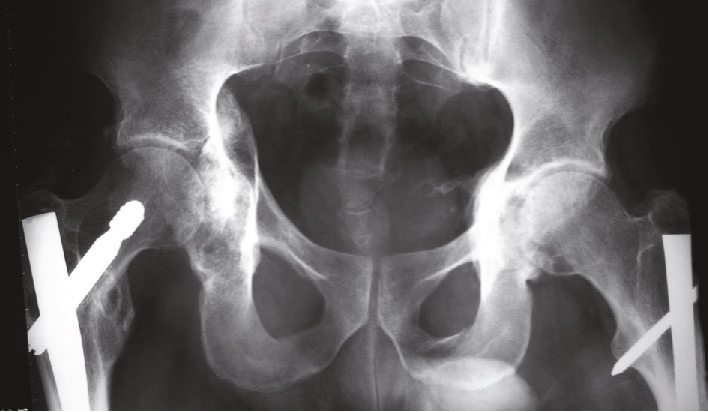
Follow-up X-ray at 10 months postsurgery.

**Figure 4:**
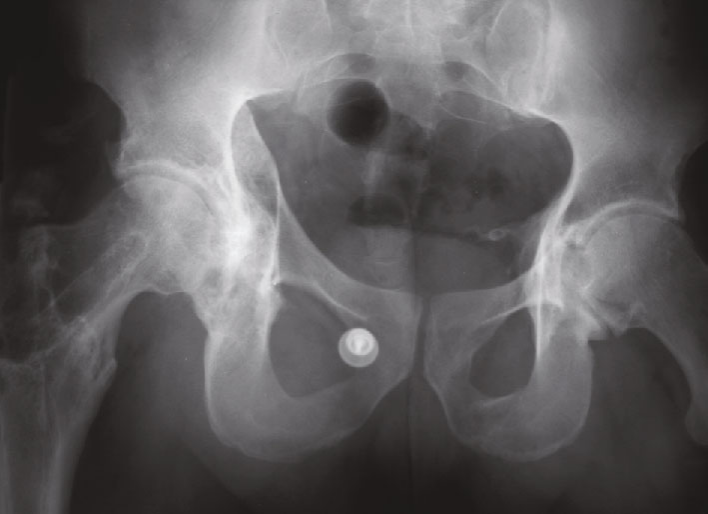
Follow-up X-ray at 2 years postsurgery.

To ensure the sectioned tendons healed, the patient was suspended in a special bed for 3 weeks and weight-bearing was authorised on day 45, i.e., almost 3 months after the initial accident, as the patient had bilateral femoral fractures. The patient received radiological and clinical follow-up for 2 years postsurgery (Figures 3 and 4), at which time Mr. D. returned to work as a delivery driver, following removal of the 2 gamma nails.

**Figure 5:**
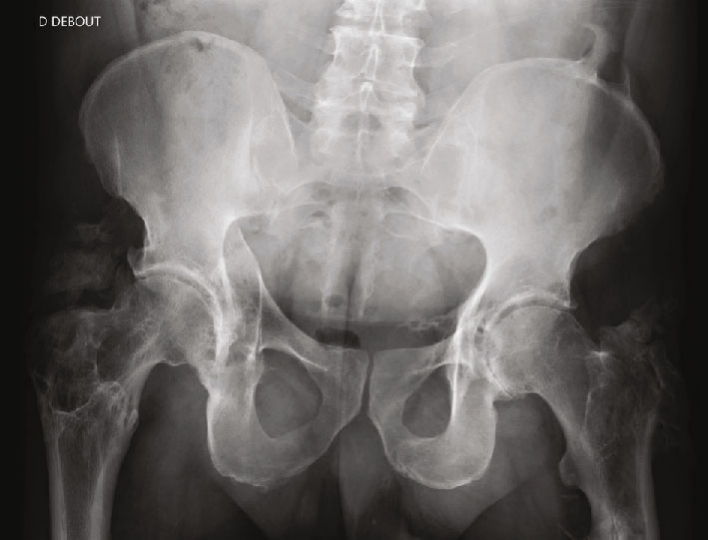
Follow-up X-ray over 20 years postsurgery (AP view).

**Figure 6:**
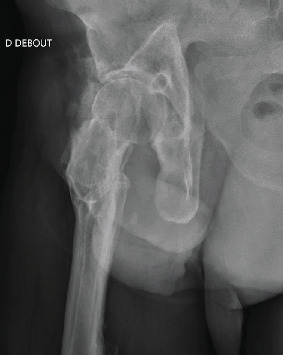
Lateral view of the X-ray in Figure 5.

Over 20 years later, the patient, still working as a delivery driver, consulted for the left hip pain, linked to early-stage osteoarthritis. The right hip was still pain-free, and the ranges of motion were as follows: flexion 100°, extension 180°, internal rotation 10°, external rotation 30°, abduction 30°, and adduction 10°. Radiologically, there was no visible osteoarthritis of the hip, and the intra-acetabular graft was fully integrated (Figures 5 and 6). Other than an asymptomatic calcification in the gluteus medius (due to the nail? the operation?), there were no surgical sequelae.

## 5. Discussion

Acetabular fracture malunions are relatively common following orthopaedic or surgical treatment. Treating them is difficult, and there is a lack of published data on the subject. The treatment most often recommended over the course of the past 20 years has been total hip arthroplasty (THA) [3, 4, 5]. Although this indication is perfectly justified in mature adults with hip problems, it is debatable in young adults, especially if the malunion can be treated without causing further damage which could impact the future use of a prosthesis. We believe that the intra-acetabular buttress technique has never previously been published. The observation presented with a very long follow-up shows that this technique is perfectly viable and nonaggressive for pelvic continuity. At the time, we chose this indication intraoperatively, given that reduction of the fracture was not possible and with just standard X-rays (AP and Judet view) at our disposal. Today, with 2D and especially 3D scans, it is entirely possible for this type of fracture with acetabular protrusion and femoral head-acetabular roof incongruence, to know preoperatively if congruence will be satisfactory following reduction of the protrusion. Where this is the case, an intra-acetabular buttress with a bone bank allograft is an indication worth considering, given this particularly remarkable observation.

## 6. Conclusion

In acetabular malunion with intrapelvic protrusion, an intra-acetabular buttress is a viable alternative, given that it restores femoral head-acetabulum congruence. This observation with a long follow-up has encouraged us to promote this technique, especially as the use of modern imaging techniques allows easy identification of the ideal preoperative indications.
